# The Response in Air Quality to the Reduction of Chinese Economic Activities During the COVID‐19 Outbreak

**DOI:** 10.1029/2020GL088070

**Published:** 2020-06-05

**Authors:** Xiaoqin Shi, Guy P. Brasseur

**Affiliations:** ^1^ Max Planck Institute for Meteorology Hamburg Germany; ^2^ National Center for Atmospheric Research Boulder CO USA; ^3^ Department of CEE The Hong Kong Polytechnic University Hong Kong China

**Keywords:** air pollution, ozone, China

## Abstract

During the COVID‐19 outbreak that took place in early 2020, the economic activities in China were drastically reduced and accompanied by a strong reduction in the emission of primary air pollutants. On the basis of measurements made at the monitoring stations operated by the China National Environmental Monitoring Center, we quantify the reduction in surface PM_2.5_, NO_2_, CO, and SO_2_ concentrations in northern China during the lockdown, which started on 23 January 2020. We find that, on the average, the levels of surface PM_2.5_ and NO_2_ have decreased by approximately 35% and 60%, respectively, between the period 1 and 22 January 2020 and the period 23 January and 29 February 2020. At the same time, the mean ozone concentration has increased by a factor 1.5–2. In urban area of Wuhan, where drastic measures were adopted to limit the spread of the coronavirus, similar changes in the concentrations of PM_2.5_, NO_2_, and ozone are found.

## Key Points

- Surface measurements made at more than 800 monitoring stations show that the mean levels of PM_2.5_ and NO_2_ in northern China have decreased by approximately 35% and 60%, respectively, after the lockdown following the COVID‐19 outbreak of early 2020
- Simultaneously, the ozone concentration, a secondary pollutant responsible for severe health problems, has increased by a factor 1.5–2
- The same type of behavior was observed specifically in the city of Wuhan, where COVID‐19 outbreak was first reported

## 1 Introduction

During the COVID‐19 outbreak of February and March 2020 that disrupted dramatically the economy in China, emissions of primary pollutants due to transportation and industrial activity, including nitrogen oxides (NOx) and carbon monoxide (CO), were severely reduced in this region of the world. Observations above major cities in China made by the TROPOspheric Monitoring Instrument (TROPOMI) on board of the European Space Agency Sentinel 5P satellite and displayed by the Royal Belgian Institute for Space Aeronomy (https://www.aeronomie.be/en/news/2020/tropomi-observes-impact-corona-virus-air-quality-china) highlight a reduction in the tropospheric nitrogen dioxide (NO_2_) column of 30%–50% in early 2020 compared to the values recorded during same period in 2019 (see Figures SI‐1 and SI‐2 in the supporting information). In Wuhan, which was entirely locked down during the coronavirus outbreak, the average tropospheric NO_2_ column, which was of the order of 3 × 10^16^ molecules cm^−2^ during the 10–15 February 2019 period, was reduced to (6–7.5) × 10^15^ molecules cm^−2^ during the same period 1 year later (https://earthobservatory.nasa.gov/images/146362/airborne-nitrogen-dioxide-plummets-over-china).

One question of interest is the response of the secondary pollutants to such large reduction in the emission of primary pollutants and specifically the impact of these changes on the concentrations of surface ozone (O_3_). Measurements made in recent years at the monitoring sites of the China Ministry of Ecology and Environment (http://english.mee.gov.cn) have shown that, in response to the efforts made to reduce emissions, surface ozone has increased by typically 1 and 2 ppb per year at urban and background sites (Gao et al., 2017; Ma et al., 2016, 2019; Sun et al., 2016). Li et al. (2019) derived for the period 2013–2017 a positive trend in the daily maximum 8‐hour average (MDA8) ozoneof about 10 ppb in the megacity clusters of Beijing and Shanghai and about 2 ppb in the southern region around Guangzhou.

In this study, we analyze measurements made during the January and February 2020 period in northern China and compare them with similar observations made during the same period in 2019. We analyze more specifically the situation in two urban areas in which the economic activity has been severely reduced after 23 January 2020. Several limitations in this comparison between the time periods should be stressed. First, the level of air pollution in China has been gradually reduced (Zhang et al., 2019) as a result of sustained mitigation policies implemented in the country as part of the Clean Air Action (State Council of the People's Republic of China, 2018). Second, year‐to‐year variability in regional meteorology (dynamics, cloudiness) generates interannual variability in air quality, specifically in background ozone (Wang et al., 2019; Zhang et al., 2016), which affects our analysis. Third, the period of the COVID‐19 outbreak has overlapped with the Chinese holiday season, and this holiday period varies from year‐to‐year, which makes the comparison difficult.

The origin of the surface measurements considered in the present study is provided in section 2. We report here the changes in the surface concentrations of nitrogen oxides, carbon monoxide, sulfur dioxide (SO_2_), particulate matter (PM), and of ozone. CO is a product of residential combustion and power generation, while NOx is emitted primarily by industrial activity and transportation. It contributes to the photochemical formation of ozone during summertime, while, in polluted areas, it titrates ozone during the winter months. It also contributes to the formation of nitrate particles. SO_2_ is a product of coal burning (domestic and energy sectors) and is a precursor of sulfate particles. We focus in section 3 on the particular situation of Wuhan, which has been completely locked down after 23 January 2020. We then extend in section 4 our analysis to the capital city of Beijing and in section 5 to the entire region of northern China.

## 2 Data Description

All observational data except for those used for the analysis in Beijing are provided by the 1,641 operational stations (1,605 stations in 2019) of the China Environmental Observation Network operated by the China National Environmental Monitoring Center (http://www.cnemc.cn/en/). The hourly measured concentrations include observations of PM_2.5_ (PM with dynamical diameter less than 2.5 μm), PM_10_, NO_2_, O_3_, SO_2_, CO, and Air Quality Index (AQI). In this study, the northern China geographical domain extends from longitudes 106°E to 125°E and from latitudes 29°N to 41°N (Figure SI‐3). It includes 853 stations (830 stations in 2019). The data representatives of the city of Wuhan are provided by the 10 monitoring stations shown in the Figure SI‐3. The observation in Beijing was provided by the Beijing Municipal Environmental Monitoring Center (http://www.bjmemc.com.cn/) including 34 stations (Figure SI‐3). The same variables were measured hourly. The concentrations values reported here are expressed in mass density. For conversion in volume mixing ratio, use 1 μg m^−3^ = 0.484 ppbv for O_3_, 0.505 ppbv for NO_2_, and 0.363 ppbv for SO_2_. In the case of CO, 1 m*g m^−3^ = 0.830 ppmv (surface pressure of 1,013 hPa and temperature of 10°C)*.

## 3 The Situation in the Urban Area of Wuhan

**Figure 1:**
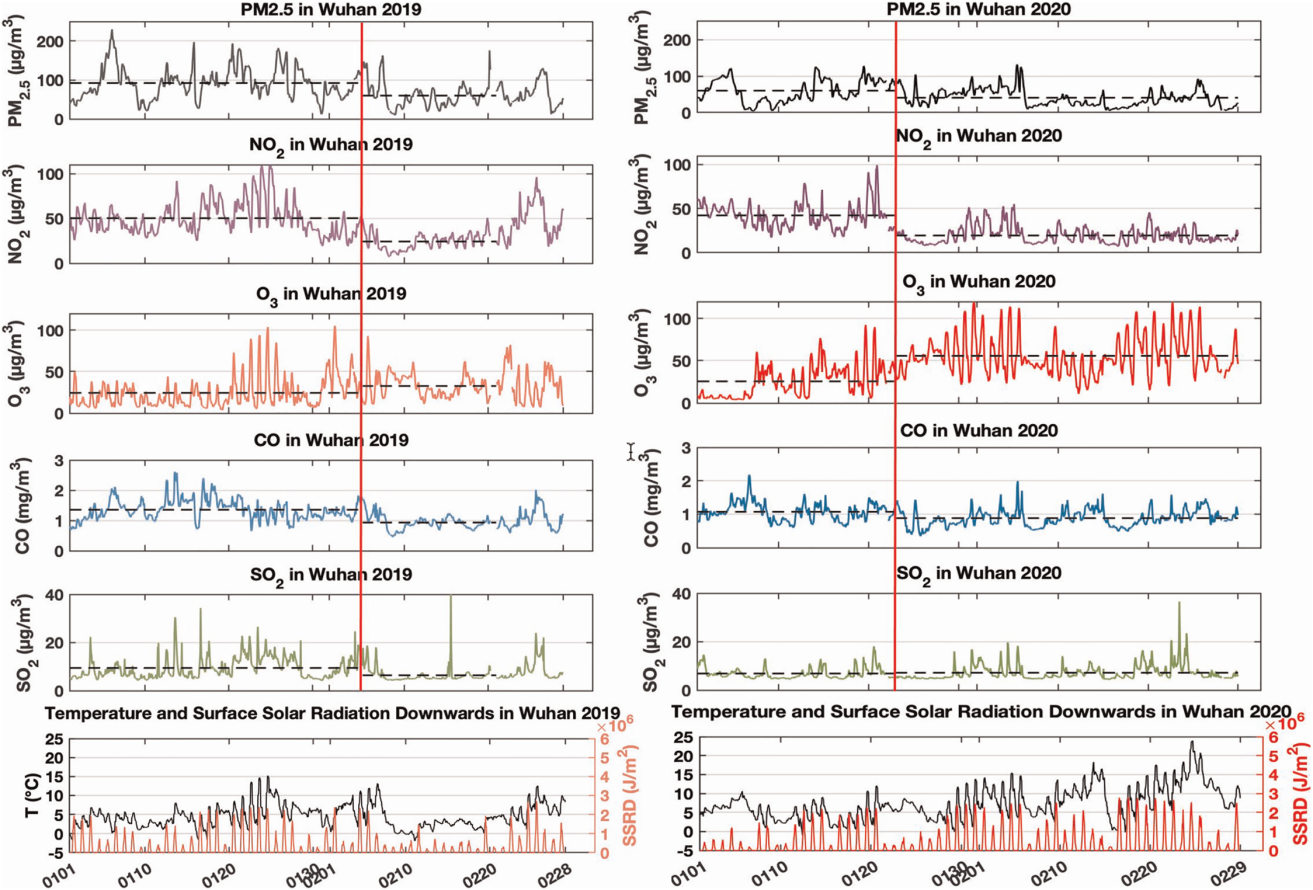
Left panel: Evolution of the mean concentration of PM_2.5_, NO_2_, O_3_, CO, and SO_2_ (all in μg m^−3^ except in mg m^−3^ for CO) recorded by the monitoring stations in the urban area of Wuhan from 1 January 2019 to 28 February 2019. The vertical red line corresponds to the beginning of the Spring Festival on 5 February 2019. The horizontal dash lines indicate the averages of the quantities before and after this date. Right panel: Same as on the left panel, but for the period 1 January 2020 to 29 February 2020. The red vertical line indicates the day (23 January 2020) during which the lockdown of Wuhan was implemented by the Chinese authorities. The horizontal dash lines show the mean of the represented quantities before and after this date. The data of temperature and surface solar radiation downwards (SSRD) are from the Copernicus Climate Change Service (C3S) (n.d.).

Since the earliest and most drastic measures to reduce people's exposure to the COVID‐19 were taken in the city of Wuhan, where the coronavirus outbreak was first reported, we reproduce in Figure 1 the evolution of the surface concentration of PM_2.5_, NO_2_, O_3_, CO, and SO_2_ in this area, from the beginning of January to the end of February 2019 and 2020. The vertical red line on the 2020 panel indicates the timing of the activities' interruption (23 January 2020) imposed by the Chinese government. The comparison of the two situations prior to 23 January shows that, in both years, the levels of NO_2_ were comparable (about 40–50 μg m^3^), while, on the average, the levels of PM2.5, CO, and SO_2_ were slightly lower in 2020 compared to 2019. Ozone concentrations were slightly higher in 2020 than in 2019, but the variability associated with the meteorological situations makes the comparison not straightforward. A more detailed analysis would require an in‐depth examination of the differences in meteorological patterns during the different periods, which is out of the scope of the present paper.

An inspection of the 2020 curves in Figure 1 for the period following the 23 January lockdown shows a decrease in the surface concentrations of PM_2.5_, NO_2_, and CO. These three atmospheric species have been affected by the imposed interruption in automobile traffic and the reduction in industrial activity during the lockdown. The concentrations of these species after 23 January also appear to be lower than during the same period in 2019. SO_2_ does not exhibit any substantial change, however, probably because this compound is produced by coal burning for residential heating and energy production. These may not have been substantially reduced during the look‐down of the city. Ozone is increasing after January 23 and is higher than during the same period in 2019.

**Figure 2:**
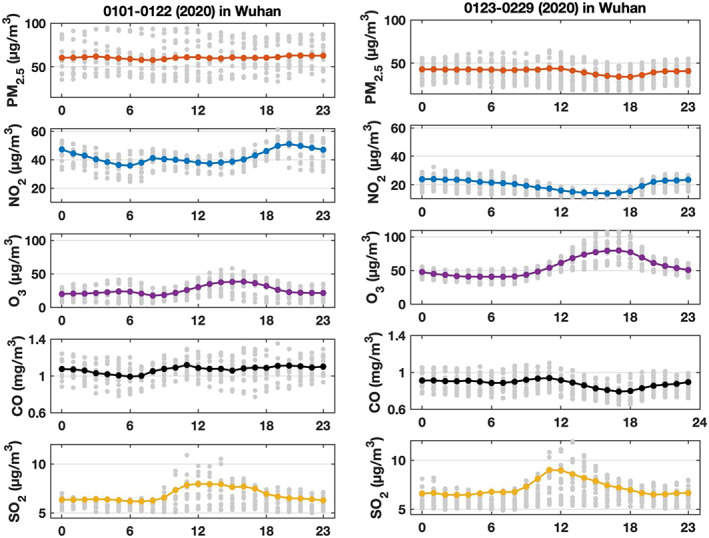
Average diurnal variation of the PM_2.5_, NO_2_, O_3_, CO, and SO_2_ concentrations (in μg m^−3^ except CO in mg m^−3^) recorded in the urban area of Wuhan: Values are for the period 1–22 January 2020 (left) and for the period 23 January to 29 February 2020 (right). The range of gray dots range from 25th percentile and 75th percentile of daily values at each hour in the specified period, averaged over all monitoring stations.

A more detailed analysis can be performed by analyzing the average diurnal variation of PM_2.5_, NO_2_, CO, SO_2_ and O_3_ for different periods and resulting from measurements at monitoring stations in Wuhan. We first compare (Figure 2) the mean of the concentrations of PM_2.5_, NO_2_, CO, SO_2_, and O_3_ for two distinct time periods: 1–22 January 2020 (before the lockdown) and from 23 January to 29 February 2020 (during the lockdown). We note that, from the first to the second period, the mean level of PM_2.5_ decreased from about 60 to 40 μg m^−3^ (−33%), that of CO from about 1.1 to 0.85 mg m^−3^ (−23%), and that of NO_2_ from 45 to 20 μg m^−3^ (−55%). At the same time, the ozoneconcentration maximum around 16:00 LT has increased from 38 to 79 μg m^−3^ (+108%). The nighttime ozone concentration was of the order of 20 μg m^−3^ before 23 January and 45 μg m^−3^ in the second period. In the case of SO_2_, we note a slight increase in the average daytime concentration, perhaps associated with enhanced residential burning (heating and cooking) during the lockdown period. In summary, in Wuhan, for all chemical species under consideration except SO_2_, there was a clear transition between the periods before and after 23 January 2020.

When we compare (see Figure SI‐4) the measured concentrations for the same period of the year (23 January to the end of February) in years 2018, 2019, and 2020, we note a substantial reduction between 2019 and 2020 in the diurnally mean concentration of the two primary atmospheric pollutants: approximately 40% in the case of PM_2.5_ and 50% in the case of NO_2_. The comparison between 2018 and 2020 for the same period of time shows a mean reduction of 38 percent in the case of PM_2.5_ and 60% in the case of NO_2_. In 2019, the mean diurnal variation of PM_2.5_ is small, while in 2020, a significant decrease in the concentration is observed in the afternoon. This decrease, also observed in the case of NO_2_ in 2020, is attributed to the expansion in the vertical of the boundary layer during daytime and the related dispersion of pollutants along the vertical. The photolysis of NO_2_ during daytime is another factor that contributes to the lower daytime concentrations. The same processes are expected to occur in 2019, but they may have been overshadowed by the fact that daytime emissions of PM_2.5_ and NOx were larger in 2019 than in 2020. In the case of ozone, the peak concentration takes place around 16:00 in both years. The magnitude of this maximum concentration, however, is a factor of 1.7 higher in 2020 compared to 2019 (79 against 46 μg m^−3^). Thus, in Wuhan ozone was substantially higher (35% to 95%) during the lockdown period of 2020 than during the same period one year earlier. It was 11%–68% higher when compared to the same period in 2018.

**Figure 3:**
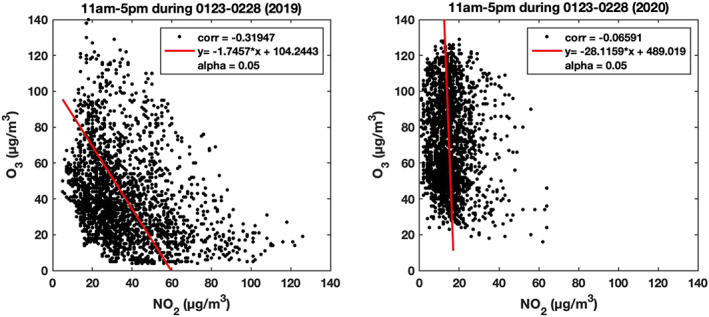
Scatterplot representing daytime ozone (11 am to 5 pm) measured at Wuhan as a function of measured nitrogen dioxide between 23 January and 28 February in 2019 (left panel) and during the same period in 2020 (right panel).

The relation between observations of daytime NO_2_ and O_3_ at all monitoring stations in Wuhan for the period 23 January to end of February in years 2019 and 2020, respectively, is exhibited in Figure 3. In the 2019 case, the concentration of surface ozone decreases substantially with increased concentration of NO_2_. When the NO emissions are sufficiently large, nitric oxide (NO) released in the atmosphere converts a large fraction of ozone into NO_2_ (Monks et al., 2015). During winter, when the concentration of NOx is high, and the level of UV radiation is low (VOC‐limited conditions), the ozone production varies inversely with the NOx concentration (Sillman et al., 1990); thus, a reduction in NOx, while all other quantities remain constant, leads to an increase of the ozoneconcentration. In the 2020 case, with lower NOx concentrations, the observed variations in the ozone concentration could be related to changes in the concentrations of VOCs and CO, in solar irradiance (cloudiness) and in meteorological variability (affecting the transport of background ozone). Unfortunately, no information is provided about the concentration of VOC and its probable reduction during the Chinese lockdown.

Another factor to be taken into consideration is the impact on ozone of the reduction in the atmospheric concentration of aerosol particles. Li et al. (2019), in their study to explain the ozoneincrease observed in China during the last years, highlight that aerosols scavenge HO_2_ and NOx radicals that otherwise would produce ozone, particularly during summertime. Tie et al. (2005) estimate the importance of heterogenous reactions on the atmospheric abundance of tropospheric oxidants including ozone. Using a three‐dimensional model, they show that the loss of the HO_2_ radical on the surface of sulfate aerosols substantially reduces the formation of ozone, particularly under high NOx levels. In their model, an additional, yet less intense reduction in ozone results from the effect of aerosols on radiative transfer with impacts on the photolysis rates of species like ozone and NO_2_. In eastern China where the aerosol load is high, Tie et al. (2005) estimate that heterogeneous reactions lead to a 60% decrease in HO_2_ and a 15% decrease in ozone relative to a case where these reactions are ignored. The substantial reduction in PM2.5 levels observed following the COVID‐19 outbreak in early 2020 could therefore have led to some, but not all of the observed increases in ozone concentration since the atmospheric concentration of HO_2_ is relatively low in winter.

## 4 The Situation in the Urban Area of Beijing

The measures taken in different urban centers of China were not necessarily as strict as in Wuhan. Here, we examine the situation in the capital city of Beijing and note that the evolution of concentrations of PM_2.5_, NO_2_, O_3_, CO, and SO_2_ (average of 34 stations) from 1 January to 28 February 2019 and 2020, shown in Figure SI‐5, is characterized by substantial variability including the occurrence of two pollution events, one between 25 January and 1 February 2020 and the second one between 8 and 14 February 2020. What is striking, however, is the significant decrease occurring in the level of NO_2_ (−40%) after 23 January 2020 as well as the concomitant increase in the concentration of ozone(+50%).

When examining the mean diurnal variation of PM_2.5_, NO_2_, and CO for the periods 1–22 January 2020 and 23 January to 29 February 2020 (Figure SI‐6), the difference between the two periods is less pronounced than in Wuhan. This could highlight that the slowdown in economic activities has been less dramatic in the capital city of Beijing than in the locked down city of Wuhan. It could also be related to specific meteorological differences between the two periods.

On the average, the level of PM_2.5_ was somewhat higher during the locked down period than during the three first weeks of January, primarily because of the occurrence of the two pollution peaks (with PM2.5 concentrations higher than 200 μg m^−3^).

## 5 Regional Analysis for Northern China

**Figure 4:**
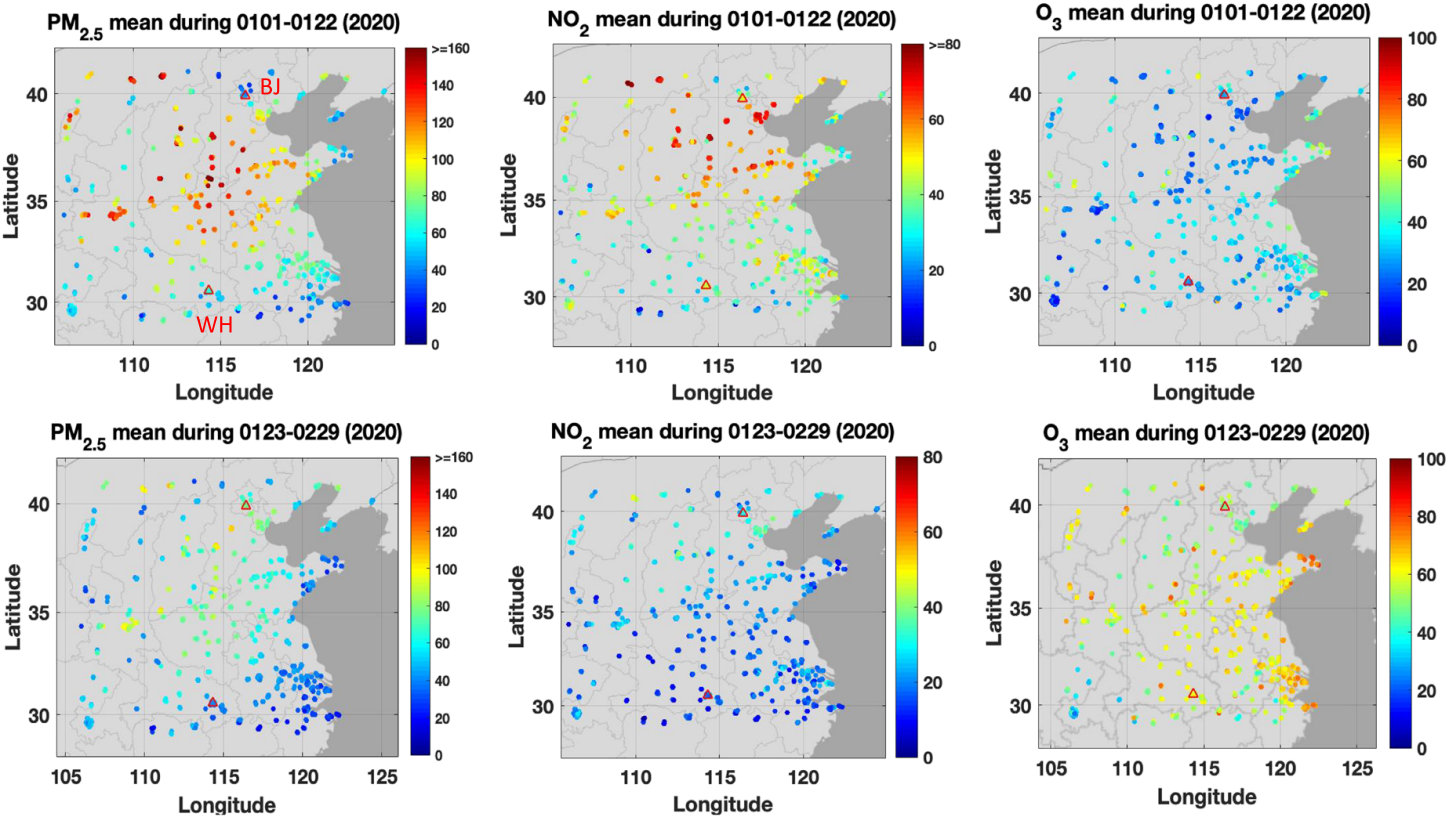
Mean concentration (μg m^−3^) of PM_2.5_ (left), NO_2_ (center), and ozone (right) in northern China. Upper panels: Averages for the period 1–22 January 2020; lower panel: Averages for the period 23 January–29 February 2020. The empty triangles show locations of the cities of Beijing (BJ) and Wuhan (WH).

We now extend our analysis to the northern part of China, which usually experiences high pollutant levels, but was strongly affected by the drastic reduction in economic activities during the COVID‐19 outbreak. Figure 4 shows the average concentrations of PM_2.5_, NO_2_ and ozone recorded at the monitoring stations of the national air pollution network. The upper panels refer to the period before the outbreak and the lower panel to the period after the outbreak. The figure clearly shows the substantial differences between the situations before and after 23 January 2020. In the case of PM_2.5_, the mean concentration levels in the geographical area south of Beijing reach more than 120 μg m^−3^ in the early weeks of January and decrease to typically 60–80 μg m^−3^ when averaged over the period 23 January to 29 February 2020. A similar reduction is observed in the case of NO_2_. The mean concentration values decrease from about 50–60 μg m^−3^ before 23 January to 20–40 μg m^−3^ after that date. The situation is different in the case of ozone. The mean surface concentrations increase from about 20–40 μg m^−3^ during the early weeks of January 2020 to 60–70 μg m^−3^after the lockdown in late January and in February. High ozone values (80–90 μg m^−3^) are noticed specifically along the coast of the east China Sea in the vicinity of Shanghai and at the tip of the Shandong Province peninsula.

**Figure 5:**
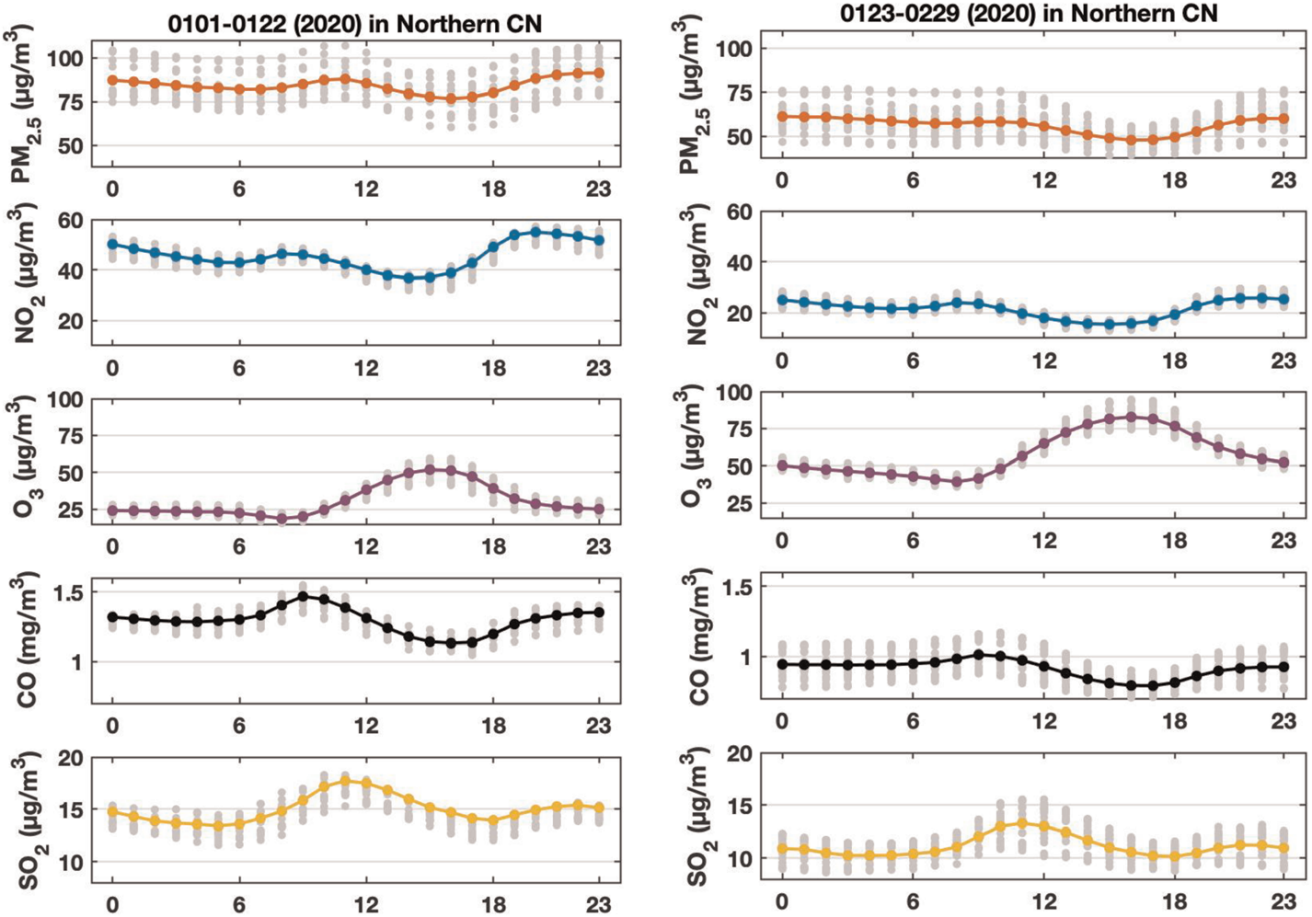
Mean diurnal variation of PM_2.5_, NO_2_, O_3_, CO, and SO_2_ concentrations (all in μg m^−3^ except CO in mg m^−3^) in northern China during the period 1 January 2020 to 22 January 2020 (left) and 23 January 2020 to 29 February 2020 (right).

The mean diurnal variation in the concentrations of PM_2.5_, NO_2_, CO, SO_2_, and ozone for the period before the lockdown (1–22 January 2020) and during the lockdown (23 January to 29 February 2020) is shown in Figure 5. Between the two time‐intervals, the concentration of PM_2.5_ has decreased from 80–90 to 50–60 μg m^−3^ and the level of NO_2_ from 35–65 to 15–25 μg m^−3^. The concentration of CO has decreased from 1.2–1.5 to 0.7–1.0 mg m^−3^ and that of SO_2_ from 14–18 to 10–13 μg m^−3^. Again, the diurnal variation in these species is influenced by the diurnal evolution of the planetary boundary layer. In the particular case of NO_2_, daytime photolysis also contributes to the lower daytime concentration of this gas and leads to the formation of ozone, whose concentration reaches a maximum around 16:00 LT. Between the two time periods under consideration, the mean ozoneconcentration has increased from 20–55 to 40–80 μg m^−3^, with similar diurnal cycles.

## 6 Conclusions

The analysis of the surface concentration of primary and secondary species measured at the monitoring stations operated in northern China reveals a strong transition in air pollution as one crosses the date of the lockdown imposed to China in response to the COVID‐19 outbreak. When the averages of the data gathered by more than 800 stations before and during the lockdown are compared, we find that the mean levels of PM_2.5_ and NO_2_ in northern China have decreased by approximately (29 ± 22%) and (53 ± 10%), respectively. The ozone concentrations have increased by a factor 2.0 ± 0.7. These results are consistent with the recent findings of Huang et al. (2020) and Wang et al. (2020). In the city of Wuhan, where the commercial and industrial activity was put to a complete hold on 23 January 2020, PM_2.5_ and NO_2_ concentrations measured at 10 local monitoring stations decreased by (31 ± 6%) and (54 ± 7%), respectively. Ozone concentrations increased by a factor 2.2 ± 0.2. These observations suggest that, as China is reducing its emissions of primary species such as NOx and SO_2_, as part of its efforts to reduce air pollution, wintertime ozone could increase substantially. This seems to be corroborated by the increase in ozone reported in recent years at different locations in China while the levels of SO_2_ and NOx were decreasing in response to air pollution mitigation measures (Li et al., 2020). Therefore, the substantial reduction in NOx and PM_2.5_, as observed during the Chinese lockdown, may not have been sufficient to avoid an ozone penalty.

## Supporting information

Supporting Information S1

## Data Availability

The data from the Chinese monitoring stations used in the present study can be obtained from https://data.4tu.nl/repository/uuid:30d132f6-e82e-473c-a8d7-e7855b188aad. This paper is dedicated to Dr. Andreas Hilboll, an atmospheric scientist at Bremen University who became affected by the COVID‐19 and passed away a few days later at age 42.
