# Supplementary material for: The Response in Air Quality to the Reduction of Chinese Economic Activities During the COVID‐19 Outbreak
Source: Geophys Res Lett. 2020 Jun 5;47(11):e2020GL088070. doi: 10.1029/2020GL088070 (PMC7267158; doi:10.1029/2020GL088070)
Supplement: Supplementary file 1 — Supporting Information S1 [file GRL-47-0-s001.docx]

**Supplementary Information**

We present here supplementary information that supports the discussion in our paper, including space observations of the NO_2_ column by the TROPOMI instrument (Figures SI-1 and SI-2) as well as the location of the monitoring stations (Figure SI-3) whose measurements were used in our study. We also show (Figure SI-4) the diurnal variation in the averaged concentration ratio of PM_2.5_, NO_2_ and O_3_ in Wuhan between 2018 and 2020 and between 2019 and 2020 (period of 23 January to the end of February). Finally, we provide for the urban area of Beijing more specific information about the evolution of the concentration of pollutants during January - February 2019 and 2020 (Figure SI-5). We also compare (Figure SI-6) the mean diurnal variation of these chemical species for the period 1 January to 22 January 2020 and January 23 to February 29 2020.


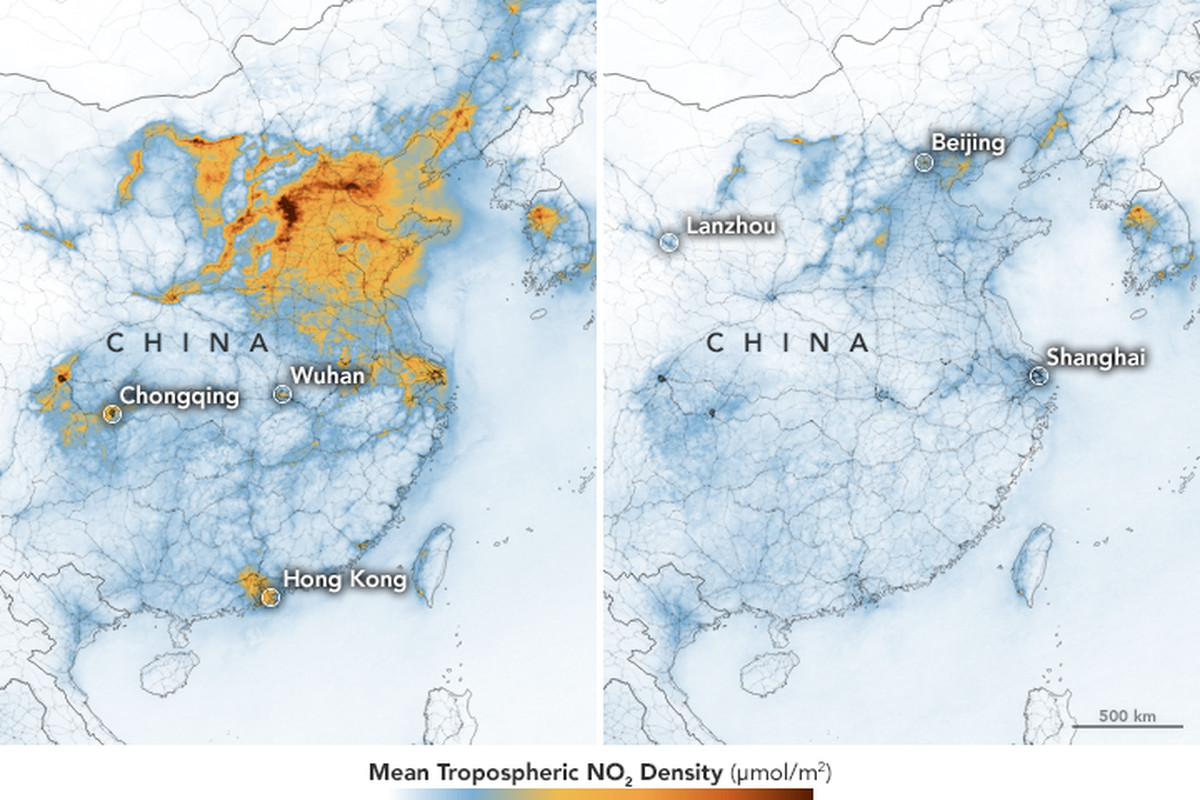


**Figure SI-1**: Vertical column of nitrogen dioxide [μmol m^-2^] measured in China by the TROPOMI instrument averaged over the period 1-20 January 2020 (left) and 10-25 February 2020 (right). From NASA: https://earthobservatory.nasa.gov/images/146362/airborne-nitrogen-dioxide-plummets-over-china


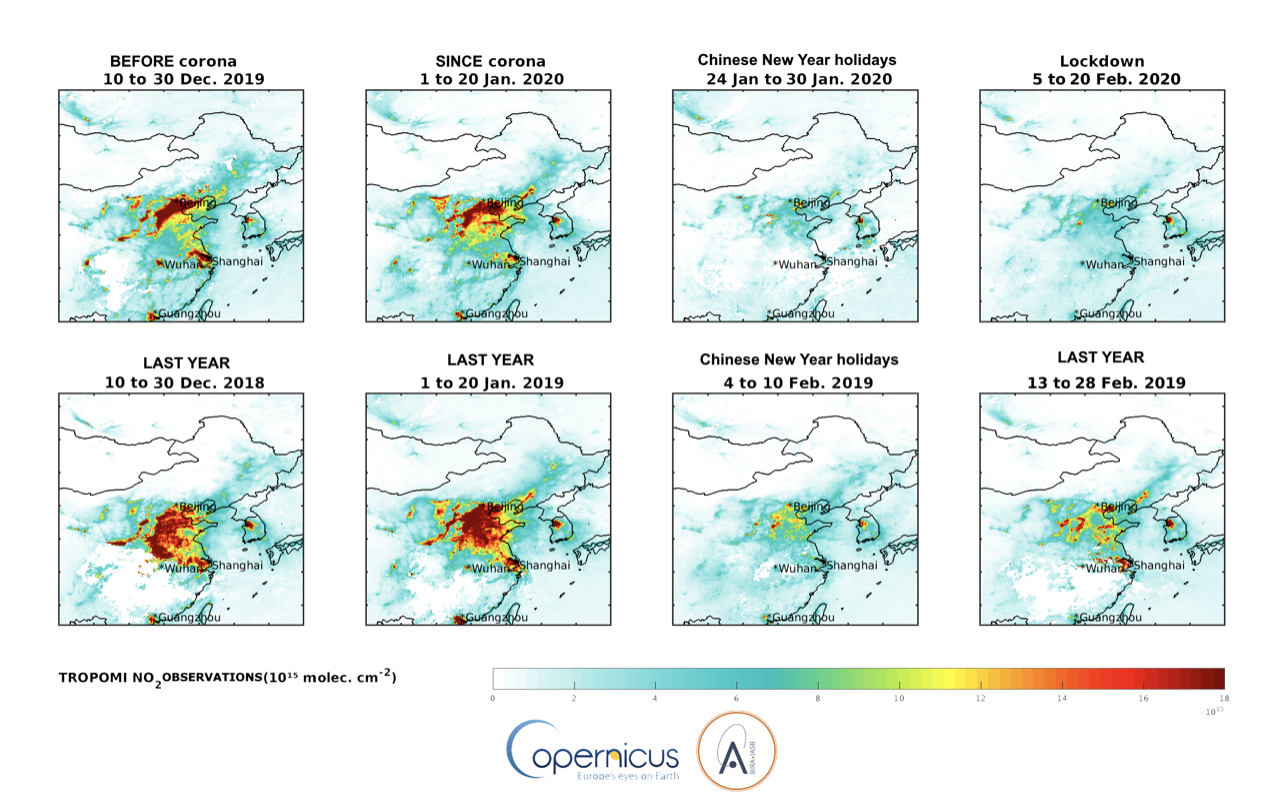


**Figure SI-2**. Vertical column of nitrogen dioxide measured in China by the TROPOMI instrument during different periods from December 2018 to February 2020 released by the Belgian Royal Institute for Space Aeronomy.

Reproduced from https://www.aeronomie.be/en/news/2020/tropomi-observes-impact-corona-virus-air-quality-china

**Figure SI-3**. Geographical distribution of the stations considered in our analysis of the measurements of northern China (upper panel), of the urban region of Wuhan (lower panel, left), and of the capital city of Beijing (lower panel, right).

**Figure SI-4.** Change in the averaged diurnal cycle of PM_2.5_, NO_2_ and O_3_ [μg m^-3^] in the city of Wuhan during the period 23 January to 28 February 2020 compared to the same period in 2018 (left) and 2019 (right).

F**igure SI-5.** Left panel: Evolution of the mean concentration of PM2.5, NO2, O3, CO, and SO2 (all in μg m−3 except in mg m−3 for CO) recorded by the monitoring stations in the urban area of Beijing from 1 January 2019 to 28 February 2019. The vertical red line corresponds to the beginning of the Spring Festival on 5 February 2019. The horizontal dash lines indicate the averages of the quantities before and after this date. Right panel: Same as on the left panel, but for the period 1 January 2020 to 29 February 2020. The red vertical line indicates the day (23 January 2020) during which the lockdown of Wuhan was implemented by the Chinese authorities. The horizontal dash lines show the mean of the represented quantities before and after this date. The data of temperature and surface solar radiation downwards (SSRD) are from the Copernicus Climate Change Service (C3S).

**Figure SI-6.** Mean diurnal variation of the PM_2.5_, NO_2_, O_3_, CO and SO2 concentrations [all in μg m^-3^ except in mg m^-3^ for CO] recorded in the urban area of Beijing. Values for 1 to 22 January 2020 (left) and for 23 January to 29 February 2020 (right).
